# Metronomic chemotherapy (mCHT) in metastatic triple-negative breast cancer (TNBC) patients: results of the VICTOR-6 study

**DOI:** 10.1007/s10549-021-06375-5

**Published:** 2021-09-21

**Authors:** M. E. Cazzaniga, I. Vallini, E. Montagna, D. Amoroso, R. Berardi, A. Butera, K. Cagossi, L. Cavanna, M. Ciccarese, S. Cinieri, E. Cretella, E. De Conciliis, A. Febbraro, F. Ferraù, A. Ferzi, A. Baldelli, A. Fontana, A. R. Gambaro, O. Garrone, V. Gebbia, D. Generali, L. Gianni, F. Giovanardi, A. Grassadonia, V. Leonardi, P. Marchetti, S. Sarti, A. Musolino, M. Nicolini, C. Putzu, F. Riccardi, D. Santini, S. Saracchini, M. G. Sarobba, M. G. Schintu, G. Scognamiglio, P. Spadaro, C. Taverniti, D. Toniolo, P. Tralongo, A. Turletti, R. Valenza, M. R. Valerio, P. Vici, P. Di Mauro, V. Cogliati, S. Capici, L. Clivio, V. Torri, The VICTOR Study Group, M. E. Cazzaniga, I. Vallini, E. Montagna, D. Amoroso, R. Berardi, A. Butera, K. Cagossi, L. Cavanna, M. Ciccarese, S. Cinieri, E. Cretella, E. De Conciliis, A. Febbraro, F. Ferraù, A. Ferzi, A. Baldelli, A. Fontana, A. R. Gambaro, O. Garrone, V. Gebbia, D. Generali, L. Gianni, F. Giovanardi, A. Grassadonia, V. Leonardi, P. Marchetti, S. Sarti, A. Musolino, M. Nicolini, C. Putzu, F. Riccardi, D. Santini, S. Saracchini, M. G. Sarobba, M. G. Schintu, G. Scognamiglio, P. Spadaro, C. Taverniti, D. Toniolo, P. Tralongo, A. Turletti, R. Valenza, M. R. Valerio, P. Vici, L. Clivio, V. Torri

**Affiliations:** 1 Phase 1 Research Centre and Oncology Unit, Department of Medicine and Surgery, University of Milano-Bicocca, ASST Monza, Via Pergolesi 33, 20900 Monza, MB Italy; 2 Oncology Unit, ASST Monza, Monza, MB Italy; 3 Medical Oncology, ASST Sette Laghi Ospedale Di Circolo E Fondazione Macchi, Varese, VA Italy; 4 Medical Senology Division, IEO, Milan, Italy; 5 Medical Oncology, Ospedale Versilia, ATNO, Lido Di Camaiore, LU Italy; 6 Medical Oncology, Università Politecnica Delle Marche, AOU Ospedali Riuniti, Ancona, Italy; 7 Medical Oncology, Ospedale San Giovanni Di Dio, Agrigento, Italy; 8 Medical Oncology, Ospedale Ramazzini, Carpi, Italy; 9 Medical Oncology, Azienda Ospedaliera Piacenza, Piacenza, Italy; 10 Medical Oncology, Ospedale Vito Fazzi, Lecce, Italy; 11 Medical Oncology, ASL Brindisi, Brindisi, Italy; 12 Medical Oncology, Ospedale Bolzano, Bolzano, Italy; 13 Medical Oncology, ASL Asti, Asti, Italy; 14 Medical Oncology, Ospedale S. Cuore di Gesù Fatebenefratelli, Benevento, Italy; 15 Medical Oncology, Osp Taormina, Taormina, Italy; 16 Medical Oncology, A.S.S.T. Ovest Milanese Legnano, Legnano, Italy; 17 Medical Oncology, Ospedale San Salvatore, Pesaro, Italy; 18 Medical Oncology 2, Az. Ospedaliero-Universitaria Pisana, Pisa, Italy; 19 Medical Oncology, ASST Fatebenefratelli Sacco, Milano, Italy; 20 Breast Unit Medical Oncology, A.O. S. Croce e Carle, Cuneo, Italy; 21 Medical Oncology, Ospedale La Maddalena, Palermo, Italy; 22 Medical Oncology, Istituti Ospitalieri Cremona, Cremona, Italy; 23 Medical Oncology, Azienda USL Romagna, U.O. di Oncologia Rimini, Cattolica, Italy; 24 AUSL IRCCS Reggio Emilia Provincial Oncology Unit, Reggio Emilia, Italy; 25 Medical Oncology, P.O. SS Annunziata -ASL2 Lanciano-Vasto, Chieti, Italy; 26 Medical Oncology, Ospedale Civico, Palermo, Italy; 27 Medical Oncology, A.O. Sant’Andrea, Roma, Italy; 28 IRCCS Istituto Romagnolo per lo studio dei Tumori (IRST) “Dino Amadori”, 47014 Meldola, Italy; 29 Department of Medicine and Surgery, Medical Oncology and Breast Unit, University of Parma and University Hospital of Parma, Parma, Italy; 30 Medical Oncology, A. Ospedaliera-Universitaria, Sassari, Italy; 31 Medical Oncology, A. Ospedaliera Antonio Cardarelli, Napoli, Italy; 32 Medical Oncology, Università Campus Bio-Medico, Roma, Italy; 33 Medical Oncology, Az. Osp. Santa Maria degli Angeli, Pordenone, Italy; 34 Medical Oncology, Ospedale San Francesco, Nuoro, Italy; 35 Medical Oncology, Osp Giovanni Paolo II, Olbia, Italy; 36 Medical Oncology, Ospedale Valduce, Como, Italy; 37 Medical Oncology, Casa di Cura Villa Salus-Messina, Messina, Italy; 38 Medical Oncology, A.O.U. Città della Salute e della Scienza, Osp. Molinette, Torino, Italy; 39 Medical Oncology, ASST Rhodense, Ospedale di Circolo Rho, Rho, Italy; 40 Medical Oncology, Osp. Umberto I, Siracusa, Italy; 41 Medical Oncology, P.O. Martini, Torino, Italy; 42 Medical Oncology, P.O. Vittorio Emanuele, Gela, Italy; 43 Department of Discipline Chirurgiche, Oncologiche e Stomatologiche (DICHIRONS), Medical Oncology, A.O.U. Policlinico Paolo Giaccone, Palermo, Italy; 44 Phase IV trials, IRCCS, INT Regina Elena, Rome, Italy; 45 Oncology Department, IRCCS Mario Negri Institute, Milan, Italy

**Keywords:** Metronomic chemotherapy, Triple-negative breast cancer, Vinorelbine, Cyclophosphamide, Capecitabine, Methotrexate

## Abstract

**Purpose:**

Triple-negative breast cancer (TNBC) represents a subtype of breast cancer which lacks the expression of oestrogen receptor (ER), progesterone receptor (PR) and human epidermal growth factor receptor-2 (HER2): TNBC accounts for approximately 20% of newly diagnosed breast cancers and is associated with younger age at diagnosis, greater recurrence risk and shorter survival time. Therapeutic options are very scarce. Aim of the present analysis is to provide further insights into the clinical activity of metronomic chemotherapy (mCHT), in a real-life setting.

**Methods:**

We used data included in the VICTOR-6 study for the present analysis. VICTOR-6 is an Italian multicentre retrospective cohort study, which collected data of metastatic breast cancer (MBC) patients who have received mCHT between 2011 and 2016. Amongst the 584 patients included in the study, 97 were triple negative. In 40.2% of the TNBC patients, mCHT was the first chemotherapy treatment, whereas 32.9% had received 2 or more lines of treatment for the metastatic disease. 45.4% out of 97 TNBC patients received a vinorelbine (VRL)-based regimen, which resulted in the most used type of mCHT, followed by cyclophosphamide (CTX)-based regimens (30.9%) and capecitabine (CAPE)-based combinations (22.7%).

**Results:**

Overall response rate (ORR) and disease control rate (DCR) were 17.5% and 64.9%, respectively. Median progression free survival (PFS) and overall survival (OS) were 6.0 months (95% CI: 4.9–7.2) and 12.1 months (95% CI: 9.6–16.7). Median PFS was 6.9 months for CAPE-based regimens (95% CI: 5.0–18.4), 6.1 months (95% CI: 4.0–8.9) for CTX-based and 5.3 months (95% CI: 4.1–9.5) for VRL-based ones. Median OS was 18.2 months (95% CI: 9.1-NE) for CAPE-based regimens and 11.8 months for VRL- (95% CI: 9.3–16.7 and CTX-based ones (95%CI: 8.7–52.8). Tumour response, PFS and OS decreased proportionally in later lines.

**Conclusion:**

This analysis represents the largest series of TNBC patients treated with mCHT in a real-life setting and provides further insights into the advantages of using this strategy even in this poor prognosis subpopulation.

## Introduction

Triple-negative breast cancer (TNBC) represents a subtype of breast cancer which lacks the expression of oestrogen receptor (ER), progesterone receptor (PR) and human epidermal growth factor receptor-2 (HER2): TNBC accounts for approximately 20% of newly diagnosed breast cancers and is associated with younger age at diagnosis, greater recurrence risk and shorter survival time [1]. Due to its molecular features, the main therapeutic options do not include endocrine or targeted therapy, but are limited to conventional chemotherapy [2], antibody–drug conjugates [3] and immunotherapy [4].

In this context, metronomic chemotherapy (mCHT) may represent a promising therapeutic strategy in the metastatic setting: it refers to the repeated administration of low doses of a chemotherapy agent to maintain prolonged and active plasma concentrations and to provide a favourable toxicity profile [5]. It is proposed that mCHT does not only have a direct antitumour effect but could exert its primary action on tumour microenvironment by inhibiting angiogenesis and promoting immune response. Valid and convincing preclinical and clinical data demonstrated mCHT efficacy for breast cancer treatment. For this reason, mCHT has been included in ABC-ESMO guidelines since 2017.

In vitro studies showed that mCHT inhibits the formation of new vessels through the induction of anti-angiogenic factors, like thrombospondin-1 [6], and to induce cell death by promoting apoptosis and/or autophagy in TNBC [7]. Furthermore, immune system stimulation is considered another mechanism of action of mCHT, as there is evidence that this strategy could increase cytotoxic T cells and reduce both regulatory T cells and myeloid-derived suppressor cells (MDSCs) compared to conventional chemotherapy [8].

Clinical studies in advanced breast cancer patients, evaluating the metronomic administration of one or more agents are encouraging, showed a response rate around 30–44% and a clinical benefit rate of approximately 70% [9–11]. VICTOR-6 is a multicentre retrospective cohort study, which collected data of metastatic breast cancer (MBC) patients who have received mCHT between January 2011 and December 2016 in 43 Italian Oncology sites. The centres were selected on the basis of their representativeness of the Italian distribution of oncological centres, their status as University or community Hospitals and the number of cases treated per year (more than 150 new cases of breast cancer per year). Aim of the present analysis is to provide further insights on the clinical activity of mCHT in metastatic TNBC patients.

## Methods

### Study design

We identified all the TNBC cases enrolled into the VICTOR-6 study, as planned for this pre-specified analysis.

The main study [12] obtained the approval of all the Ethical Committees. All patients provided written informed consent, if still alive at the moment of data collection. Data were collected via electronic database. Baseline information included patient’s age at metastatic diagnosis, breast cancer biological information, (histology, HR and HER status), date and site of first relapse, type of medical treatment for first metastasis, number and type of treatments received before mCHT number and sites of metastases at mCHT administration. For each patient, physicians were requested to provide a fully comprehensive description of the type (endocrine/chemotherapy) and number of treatments performed prior to mCHT therapy.

Eligibility criteria and treatment plan have been described in the main paper.

### Clinical outcomes

All measures of clinical outcomes were based on the physician’s evaluation. Primary endpoint of this analysis is to describe the clinical activity of mCHT in terms of ORR and DCR; PFS, OS, safety are secondary endpoints, as in the main paper. Patients who had not progressed, or had died were censored at the data cut-off date (October, 2017).

### Statistical considerations

Demographic data, patients’ baseline characteristics and disease, plus treatment information were summarized with standard summary statistics (mean standard deviation and range for continuous data, relative and absolute frequencies for categorical data). Relationships of these variables with response were analysed by mead of a Mantel–Haenzel. Time to event analysis was described by Kaplan–Meier approach and association with baseline characteristic was analysed by stratified log-rank test and proportional hazard model.

Univariate and multivariate logistic analyses were used for estimating the association of selected basal characteristics and treatment with response. Odds ratio and relative 95% confidence interval (CI) were used as summary statistics. The number of patients was calculated in order to obtain a quite precise description of chosen statistics and a good fit with the Cox model.

The data were statistically analysed using SAS version 8 (SAS Institute Inc., Cary, NC).

## Results

### Patient and tumour characteristics

Between January 2011 and December 2016, we retrospectively retrieved clinical data of 584 metastatic breast cancer patients treated with mCHT, of whom 97 (16.6%) patients were classified TNBC, according to ASCO-CAP guidelines [13], who are the population object of this analysis.

At primary diagnosis, main tumour characteristics were ductal histology (88.2%), pT2 stage (53.6%), pN1 stage (31.9%); 21 (21.6%) patients were metastatic at diagnosis. Grading was G3 in 66% of the tumours.

Median follow-up time was 37.6 months (13.3–41.8). Median age at the time of diagnosis was 67 years (30–88). Median Disease Free Interval (DFI) was 17 months (0–288).

At the time of first relapse, 41.2% of the patients had 2 or more metastatic sites; bone was the main site involved (36, 37.1%), but brain metastases were present in 5.2% of the patients.

At the beginning of mCHT, the median age of the patients was 68 years (30–88), most of them had an ECOG PS of 0 (53.6%) or 1 (32.9%). Almost half of the patients had 2 or more metastatic sites (45.4%); main sites of metastases were bone (41.2%) and lung (38.1%). Central Nervous System (CNS) involvement was present in 10.3% of the patients. Table 1 summarizes patients’ and disease characteristics at first relapse and at the time of mCHT start.

**Table 1:** Patients and disease characteristics at diagnosis of first relapse and at the time of mCHT start

	At first relapse N (%)	At mCHT start
Median age (min–max)	67 (30–88)	68 (30–88)
PS (ECOG)		
0	NA	52 (53.6)
1	NA	32 (32.9)
> 2	NA	13 (13.4)
No. of metastatic sites		
0*	5 (5.2)	7 (7.2)
1	52 (53.6)	46 (47.4)
> 2	40 (41.2)	44 (45.4%)
Sites of metastatic disease		
Bone	36 (37.1)	40 (41.2)
Soft tissue	25 (25.8)	27 (27.8)
Lung	34 (35.1)	37 (38.1)
Liver	17 (17.5)	19 (19.6)
CNS	5 (5.2)	10 (10.3)
Other	37 (38.1)	39 (40.2)
Previous treatments		
None		39 (40.2)
Chemotherapy		56 (57.7)
Endocrine therapy**		2 (2.1)
No. of previous treatments		
0		39 (40.2)
1		26 (26.8)
> 2		32 (32.9)

*NA* not available, *CNS* central nervous system

*metastasis removed

**2 patients were HR + ve at the time of first relapse, becoming TNBC at the beginning of mCHT

In 40.2% of the patients, mCHT was the first chemotherapy treatment, whereas 32.9% had received 2 or more lines of treatment for the metastatic disease.

Forty-four (45.4%) out of 97 TNBC patients received a VRL-based regimen, which resulted in the most used type of mCHT, followed by CTX-based regimens (30, 30.9%) and CAPE-based combinations (22, 22.7%). MTX-based regimens were only occasionally used (1 patient).

Most of the patients received mCHT as single-agent (84, 86.6%); 40.2% of the patients have been treated with mCHT in first-line setting (39, 40.2%). Table 2 describes the distribution of mCHT regimens, according to the main drug.

**Table 2:** Describes the types of mCHT and use as monotherapy (single agent), or as combination with other drugs

	Type of mCHT in TNBC (n = 97) N (%)	Type of mCHT in Luminal (n = 487) N (%)
VRL-based	44 (45.4)	261 (53.6)
VRL single agent	32 (72.7)	170 (65.1)
VRL + CAPE/CTX	12 (27.3)	91 (34.9)
CAPE-based	22 (22.7)	121 (24.8)
CAPE single agent	21 (95.5)	111 (91.7)
CAPE + CTX	1 (4.5)	8 (6.6)
CAPE + Other drugs	–	2 (1.7)
CTX-based	30 (30.9)	96 (19.7)
CTX single agent	30 (100)	87 (90.6)
CTX + Other drugs	–	9 (9.4)
MTX-based	1 (1.0)	9 (1.8)

## Clinical activity

Regarding clinical activity, Overall Response Rate (ORR) and DCR were 17.5 and 64.9%, respectively. Best ORRs and DCRs were observed in first-line settings (20.9 and 76.7%), whereas tumour response decreased proportionally in later lines (14.8 and 55.6% for ORR and DCR, respectively). Table 3 summarizes the clinical activity according to the line of treatment and the regimen used. At the multivariate analysis, no clinical or tumour characteristics (PS, Hormone Receptor status, number of metastatic sites, previous treatments) were associated with ORR, nor was the type of mCHT.

**Table 3:** Clinical activity in TNBC and Luminal BC, according to the line of treatment and the type of mCHT

	ORR *n*/*N* (%)	DCR *n*/*N* (%)
TNBC *N* = 97		
Overall TNBC	17 (17.5)	63 (64.9)
1st-line *N* = 43	9 (20.9)	33 (76.7)
VRL-based (*N* = 25)	8 (32)	20 (80.0)
CTX-based (*N* = 12)	–	10 (83.3)
CAPE-based (*N* = 6)	1 (16.7)	3 (50.0)
2nd-line or more (*N* = 54)	8 (14.8)	30 (55.6)
VRL-based (*N* = 19)	3 (15.8)	9 (47.5)
CTX-based (*N* = 18)	2 (11.1)	11 (61.1)
CAPE-based (*N* = 14)	3 (21.4)	9 (64.3)
MTX-based (*N* = 1)	–	1

Luminal BC *N* = 420*		
Overall Luminal	124 (29.5)	321 (76.4)
1st-line *N* = 229	81(35.6)	183 (79.9)
VRL-based (*N* = 161)	61 (37.9)	134 (83.2)
CTX-based (*N* = 15)	2 (13.3)	7 (46.7)
CAPE-based (*N* = 53)	18 (33.9)	42 (79.2)
2nd-line or more (*N* = 191)	43 (22.5)	138 (72.3)
VRL-based (*N* = 126)	29 (23.0)	88 (69.8)
CTX-based (*N* = 7)	–	2
CAPE-based (*N* = 58)	14 (24.1)	48 (82.8)
MTX-based *N* = 0	–	–

*Patients evaluable for tumour response 420 out of 487

In TNBC patients, both ORRs and DCRs resulted in lower percentages in comparison to those obtained in Luminal patients enrolled into the main study (TNBC vs Luminal—ORR: 17.5 vs 29.5%; DCR: 64.9 vs 76.4%), independently of the line of treatment. Table 3 summarizes the clinical activity of mCHT in both TNBC and Luminal populations, according to the line of treatment and the type of regimen.

Median PFS and OS were 6.01 months (95% CI: 4.9–7.2) and 12.1 months (95% CI: 9.6–16.7) in the whole TNBC population, respectively.

**Fig. 1:**
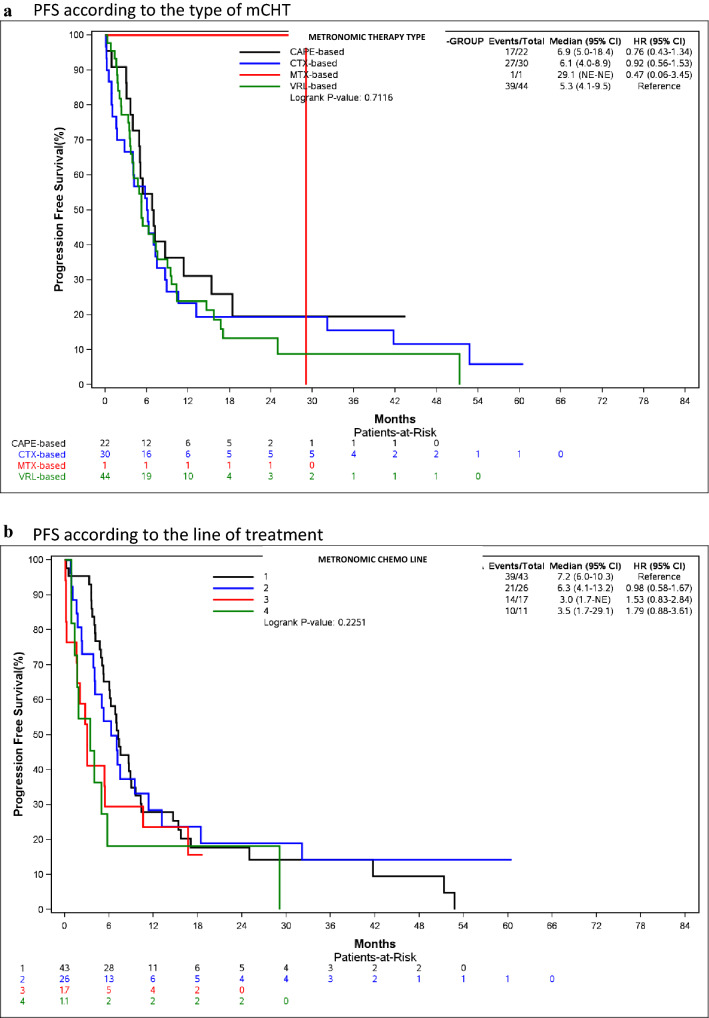
Progression-Free-Survival of mCHT regimens. **a** PFS according to the type of mCHT, **b** PFS according to the line of treatment

Median PFS was 6.9 months for CAPE-based regimens (95% CI: 5.0–18.4), 6.1 months (95% CI: 4.0–8.9) for CTX-based and 5.3 months (95% CI: 4.1–9.5) for VRL-based ones (Fig. 1a).

As expected, the longest PFS was observed when mCHT was used in first-line setting: 7.2 months (95% CI: 6.0–10.3) vs 6.3 months (95% CI: 4.1–13.2) and 3.0 months (95% CI: 1.7-NE) for second and subsequent lines (Fig. 1b).

**Fig. 2:**
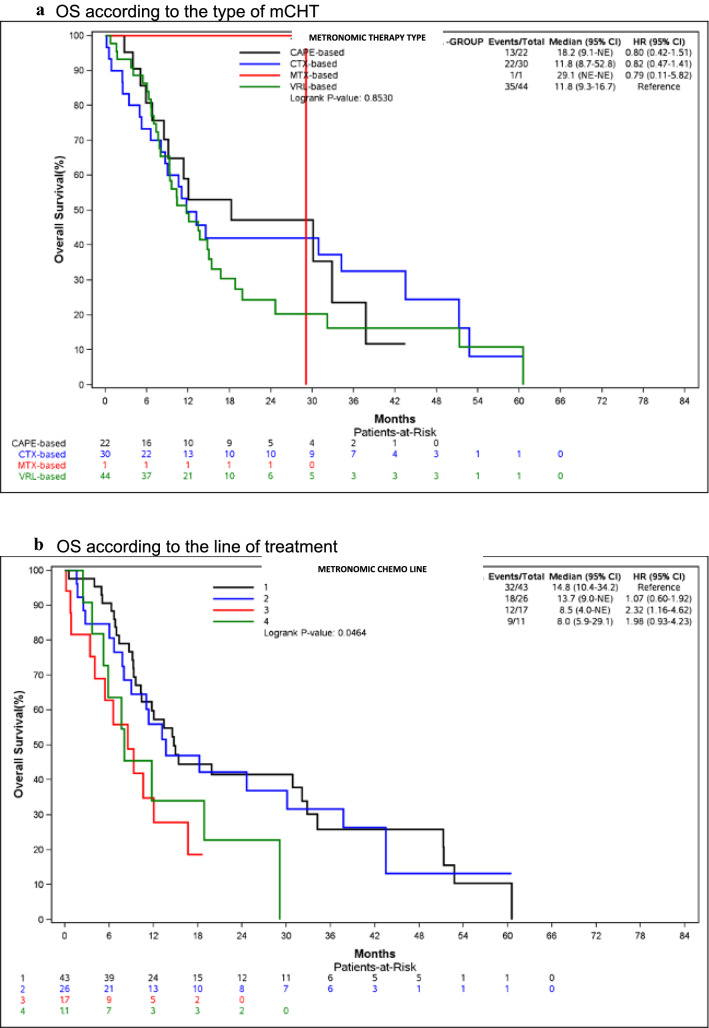
Overall Survival of mCHT regimens. **a** OS according to the type of mCHT. **b** OS according to the line of treatment

Median OS was 18.2 months (95% CI: 9.1-NE) for CAPE-based regimens and 11.8 months for VRL- (95% CI: 9.3–16.7 and CTX-based ones (95% CI: 8.7–52.8) (Fig. 2a).

Similar results were observed for OS according to the line of treatment: 14.8 months (95% CI: 10.4–34.2) vs 13.7 months (95% CI: 9.0-NE) for first- and second-line treatment, respectively (Fig. 2a). Median Survival Post Progression (SPP) was 6.3 months (95% CI: 4.5–8.7).

**Table 4:** Toxicity

Type of toxicity	Grade 1–2 *n* (%)	Grade 3–4 *n* (%)
Hematologic	10 (10.3)	8 (8.2)
Liver toxicity	2 (2.1)	3 (3.1)
Fatigue	8 (8.2)	2 (2.1)
Nausea and vomiting	10 (10.3)	1 (1.0)
Diarrhoea	11 (11.3)	–
Cutaneous (including hand-foot syndrome)	2 (2.1)	1 (1.0)
Other toxicities	7 (7.2)	1 (1.0)

### Safety

The main toxicity, any grade, was haematological (18.6%), followed by nausea and vomiting and diarrhoea (both, 11.3%) and fatigue (10.3%). Grade 3–4 haematological toxicity, mainly neutropenia, was reported in 8.2% of the cases and Grade 3–4 liver toxicity in 3.1% of the patients.

Table 4 summarizes the type of toxicity of all mCHT regimens. Discontinuation due to adverse events was observed in 12.4% of the patients.

## Discussion

The analysis presented in this paper explores the role of mCHT in advanced TNBC in a real-life setting as part of the VICTOR-6 study, an observational, retrospective study regarding the use of mCHT in the strategy of treatment of advanced breast cancer patients [12].

Median DFI in our TNBC population was 17 months (0–288), lower than that observed in the Luminal counterpart, but aligned to what reported in different studies for this subset of patients.

TNBCs are definitely biologically aggressive; shortened disease-free intervals in the adjuvant and neoadjuvant setting and a more aggressive clinical course in the metastatic setting remain the main factors contributing to this worse outcome [14, 15]. In our analysis, 5.2% of the patients at baseline and 10.3% at the time of mCHT start presented CNS involvement. Different reviews and studies reported a predilection for visceral metastasis, including lung, liver and, notably, brain metastasis; in particular, the risk for developing brain metastasis is higher for patients with TNBC than with other types of breast cancer and current estimates are that approximately 15% of TNBC patients develop brain metastasis [15].

Even though mCHT is considered by many oncologists to be a palliative treatment, 40.2% of the patients included in the present analysis received this treatment as first-line therapy, in comparison to 72 out of 487 (14.8%) Luminal BC population. Considering that the median age is approximately the same and the distribution of metastases is very similar between TNBC and Luminal BC in our collection, this finding could deserve further insights, also considering that, in the TNBC group, mCHT was used mainly as monotherapy (TNBC vs Luminal: 84/97, 86.6% vs 377/487, 77.4%).

We can argue that most oncologists consider the prognosis of these patients so desperate that they set up a therapy with purely palliative purposes from the beginning, despite international guidelines suggesting using doublets or triplets [16].

Metronomic VRL-based regimens were found to be the most used in TNBC patients (45.4%) as in Luminal BC ones (53.6%), whereas CTX-based regimens were found to be used in a higher percentage (30.9%) in TNBC patients, in comparison to Luminal ones (19.7%). Even if the small number of patients could have affected this finding, we can hypothesize that different pre-clinical results reporting the immune modulatory activity of CTX [17, 18], as well as the well-known anti-angiogenic properties, may have prompted oncologists to adopt this type of treatment.

No data are available on PDL-1 and Tumour infiltrating lymphocytes (TILs) levels in VICTOR-6 TNBC patients, as these tests were not yet routinely performed during the study period. However, taking into account the growing evidence of the immune modulatory activity of mCHT and the current possibility of using immunotherapy in TNBC, these analyses deserve further investigation in future studies.

ORR and DCR were 17.5 and 64.9%, respectively and best ORRs and DCRs were observed in first-line settings (20.9 and 76.7%). These rates are lower than those observed in Luminal tumours, being 29.5 and 76.4%, respectively (VICTOR-6, unpublished data). Median PFS and OS were 6.01 months (95% CI: 4.9–7.2) and 12.1 months (95%CI: 9.6–16.7), respectively.

Main reason for discontinuation was disease progression in the TNBC group (68%), as in Luminal patients (71.5%).

It is quite common to observe a lower activity of the same drugs or regimens in TNBC patients in comparison to their Luminal counterpart, even with standard chemotherapy: i.e. for paclitaxel, ORR ranged from 20.0 to 28.6% according to the different studies [19] in TNBC, vs > 40% in Luminal tumours [20].

Other Authors [21] reported retrospective results of mCHT with different regimens in metastatic breast cancer patients; most of them showed that ORRs were lower in TNBC subgroups in comparison to those observed in Luminal patients.

Despite the relative variety of regimens included in the present analysis, clinical activity of mCHT in this real-life setting is very close to the one observed in prospective clinical trials [11, 22].

In the VICTOR-6 study, the analysis of the TNBC patient’s subgroup [22] showed that amongst the 15 patients who received mCHT as second or further line of treatment, the DCR was 53.7%, the median duration of disease control was 7.4 months and PFS was 4.7 months. The VEX TNBC study enrolled 22 patients to receive the metronomic combination of VRL, CAPE and CTX; the ORR was 27% and the median TTP was 6.4 months [11].

Yoshimoto et al. [9] treated 45 patients, of whom only 9 were TNBC, reporting an ORR of 44.4% and a clinical benefit rate (CBR) of 57.8% in the TNBC population. The small number of patients is the main limitation of this result, which should therefore be considered with caution.

Our outcomes are supported and can be explained by emerging preclinical data. Indeed, in vitro* and *in vivo studies suggest that mCHT can exert a role in the control of both TNBC cell lines and mouse models [7, 23, 24].

Very recently, Espanol et al. [24] investigated the effect of mCHT of paclitaxel plus muscarinic agonists at low doses on TNBC cells. They reported that the addition of carbachol or arecaidine propargyl ester, a non-selective or a selective subtype 2 muscarinic receptor agonist respectively, to paclitaxel was able to reduce cell viability through the involvement of a down-regulation in the expression of ATP-binding cassette (ABC) G2 drug transporter and epidermal growth factor receptor. The Authors also demonstrated an inhibition of tumour cell migration and anti-angiogenic effects produced by those drug combinations in vitro and in vivo (in NUDE mice), respectively, providing interesting evidence about subtype 2 muscarinic receptors as therapeutic targets for the treatment of TNBC tumours.

Di Desidero et al. [23] studied the metronomic combination of topotecan and pazopanib, an antiangiogenic tyrosine kinase inhibitor (TKI), in a triple negative, primary and metastatic breast cancer orthotopic model. This treatment significantly increased antitumour activity compared to monotherapy with either drug or prolonged survival, together with a marked decrease in tumour vascularity, proliferative index and the induction of apoptosis. Significant changes in tumour angiogenesis, cancer cell proliferation, apoptosis, HIF1α levels, HIF-1 target genes and ABCG2 were found both in vitro and in tumour tissue.

Finally, our group reported that the metronomic combination of VRL and 5-Fluorouracil is able to significantly induce cell death by promoting apoptosis and/or autophagy in TNBC cell lines [7].

## Conclusion

To our knowledge, this is the largest series of TNBC patients treated with mCHT in a real-life setting. Considering that it has become more and more difficult to conduct studies in this setting of patients, mainly due to the high competitiveness of more recent targeted drugs, we strongly believe that our data can provide further insights of the real value of this strategy even in this poor prognosis subpopulation. This should be of great importance especially for TNBC patients in disadvantaged parts of the world such as LMICs, where most of the new drugs are not available for all patients.

## Data Availability

The data are available and can be sent on request.
